# Supplementary material for: Artificial Pasture Grazing System Attenuates Lipopolysaccharide-Induced Gut Barrier Dysfunction, Liver Inflammation, and Metabolic Syndrome by Activating ALP-Dependent Keap1-Nrf2 Pathway
Source: Animals (Basel). 2023 Nov 19;13(22):3574. doi: 10.3390/ani13223574 (PMC10668702; doi:10.3390/ani13223574)
Supplement: Supplementary file 1 [file animals-13-03574-s001.zip › animals-2672187-supplementary.pdf]

Table S1. Chemical composition of feed

	Diets		
**Ingredients, %**	**Grower**	**Finisher**	
Wheat	55.3	59	
Rice bran	5	4	
Corn germ meal (exp.)	4	3.2	
Dumpling powder	5	4	
Furfural residue	3	2	
Corn distiller’s grains (DDGS)	6.5	7	
Spouting germ meal	3	2	
Soybean meal (sol.)	7	6	
Peanut meal(sol.)	1.5	1	
Albumen powder	2	1.5	
Stone powder	1.1	1	
Liquid methionine	0.25	0.3	
MuLaoDa-2	1.25	2	
201/202 gunk	2.5	3	
Calcium hydrogen phosphate	0.6	1	
Chemical composition (%)	**Grower**	**Finisher**	**Ryegrass**
Crude protein	20.12	15.54	25.86
Crude fat	15.27	15.15	10.21
Ash	12.89	12.86	13.89
Dry matter	87.86	89.28	60.16
Moisture	12.14	10.82	39.86
Neutral detergent fiber	13.25	30.55	45.16
Acid detergent fiber	5.50	27.02	28.24
Calcium	1.15	1.07	0.90
Phosphorous	0.47	0.32	0.34

Table S2. **Primer sequences used for quantitative real-time PCR**

Gene	Forward primer (Sequence 5′- 3′)	Reverse primer (Sequence 5′- 3′)
NRF2	CGCCTTGAAGCTCATCTCAC	CCTCTCCTGCGTATATCCCG
iNOS	CTCATTCTCCAAGCGAACGG	GCACTCCTATCTCTGTCCCC
COX2	GGTTCTACAATGGAGAGCGC	TGTTCTTGCCACTTGAGCTG
IL-1B	CACATCACAACCCACAGCAA	CTGCCCCTTCCGTCTTCTTA
IL-6	GGAAGACCCTTGCTCTCCTT	TGGAGCCAGAAGATGAGTGG
TNF-α	GGTCCACAACGAGTTCATCC	AGGAGGAGGAGGAGATGGAG
β-actin	CAACGAGCGGTTCAGGTGT	TGGAGTTGAAGGTGGTCTCG
Keap1	CTGAACGAGGCCCTCAAGTA	CAAACTCGTAGCGGGGAATG
